# Moral approval of xenotransplantation in Egypt: associations with religion, attitudes towards animals and demographic factors

**DOI:** 10.1186/s12910-024-01013-3

**Published:** 2024-02-19

**Authors:** Gabriel Andrade, Eid AboHamza, Yasmeen Elsantil, AlaaEldin Ayoub, Dalia Bedewy

**Affiliations:** 1 Ajman University, Ajman, United Arab Emirates; 2 Al Ain University, Al Ain, United Arab Emirates; 3 Tanta University, Tanta, Egypt; 4 Arabian Gulf University, Manama, Bahrain; 5 Aswan University, Aswan, Egypt

**Keywords:** Xenotransplantation, Egypt, Pigs, Ethics, Religion, Animal welfare

## Abstract

Xenotransplantation has great potential as an alternative to alleviate the shortage of organs for donation. However, given that the animal most suited for xenotransplantation is the pig, there are concerns that people in Muslim countries may be more hesitant to morally approve of these procedures. In this study, the moral approval of xenotransplantation was assessed in a group of 895 participants in Egypt. The results showed that religiosity itself does not predict moral approval of xenotransplantation, but religious identity does, as Muslims are less likely to approve of xenotransplantation than Christians. However, the strongest predictor of moral approval of xenotransplantation was gender, with women displaying less approval. A partial mediating factor in this association was concern for animal welfare. Based on these results, some implications for public policy are discussed.

**Supplementary Information:**

The online version contains supplementary material available at 10.1186/s12910-024-01013-3.

## Introduction

Xenotransplantation is the process of transplanting organs, tissues, or cells from one species into another species, most commonly into humans. The primary goal of xenotransplantation is to address the shortage of human donor organs and potentially provide a more readily available source of organs for transplantation [1].

The process goes through various phases. First, animal donors are chosen on the basis of suitability of their organs being of similar in size and function to human organs; originally baboons and other non-human primates were used, since the 1980s pigs have been preferred in this endeavor. Second, donor animals are genetically modified to reduce the risk of immune rejection and transmission of diseases from animals to humans (xenozoonosis), donor animals are genetically modified [2]. This can involve removing or modifying specific genes responsible for producing antigens that are recognized by the human immune system. This process is known as “humanization” of the animal’s organs. Even with genetic modifications, the human recipient’s immune system can still recognize animal tissues as foreign and trigger a rejection response. To prevent this, recipients receive immunosuppressive drugs to dampen their immune response, similar to what is done in human-to-human organ transplantation [3].

Third, the donor animal organ, which has been genetically modified, is surgically transplanted into the human recipient. The specific surgical procedure varies depending on the organ being transplanted [4]. Fourth, after transplantation, the recipient is closely monitored for any signs of rejection or complications. Regular medical check-ups and immunosuppressive drug therapy adjustments may be necessary to maintain the viability of the transplanted organ [5].

While xenotransplantation is a promising alternative, some challenges remain. For example, immune rejection is still a problem, and there is a potential risk of transmitting animal viruses to humans, which must be carefully managed and monitored. Perhaps the best-known case was that of Baby Fae, who died a few weeks later after receiving a baboon’s organ, largely due to immunological complications [6]. This suggests that with xenotransplantation, there are concerns about the need for lifelong surveillance of patients after receiving the organ.

Other concerns are ethical. Xenotransplantation carries the potential risk of transmitting infectious diseases from animals to humans. Ethical concerns center on the precautionary principle, which suggests that xenotransplantation should not be pursued until the risks of zoonotic diseases are better understood and minimized [7]. Likewise, the long-term health and safety of recipients of xenotransplants are still not fully known, which raises ethical issues regarding patient safety and the potential for unforeseen complications [8].

The ethics of human-animal relations are also an issue. There are concerns about the conditions in which donor animals are raised and the potential for harm, suffering, and exploitation of these animals [9]. As with animal experimentation, there is the ethical difficulty of consent [10]. In human-to-human organ transplantation, informed consent is typically obtained from donors or their families. In xenotransplantation, there is no way to obtain informed consent from the donor animals, raising ethical questions about the use of animals for the benefit of humans without their consent [11, 12].

While these are ethical issues that are likely to affect xenotransplantation globally, there are also specific geographic areas where xenotransplantation might pose additional ethical concerns. For example, in countries with greater percentage of vegans amongst the population, or with higher sensitivity towards animal suffering, there may be hesitations about killing animals in order to use their organs.

But even more so, moral qualms about xenotransplantation may be especially salient regions where there are cultural prohibitions on pigs. Pigs are considered the most suited species for xenotransplantation [13], due to various reasons: their organs have similar sizes and functions as for humans; pigs are widely available, and they have potential for large scale reproduction; they are anatomically comparable to human beings; they can be genetically modified more effectively than some other animals; and humans have a lower risk of immune rejection of pig tissue.

Traditionally, Islamic societies have considered pigs impure animals [14]. This is done on a scriptural basis. For example, the Qur’an clearly states: “He has only forbidden to you dead animals, blood, the flesh of swine, and that which has been dedicated to other than Allah” (2:173), “Say, ‘I do not find within that which was revealed to me [anything] forbidden to one who would eat it unless it be a dead animal or blood spilled out or the flesh of swine - for indeed, it is impure” (6:145). The Islamic prohibition on the consumption of pigs is firm.

But amongst Muslim religious scholars it is still debated whether xenotransplantation from pigs would be allowed. By and large, such scholars allow for organ transplantation amongst humans, provided there is consent and conventional ethical guidelines are followed [15–17]. To the extent that xenotransplantation might be a life-saving procedure, xenotransplantation might be morally approved by Islamic ethics. But given that donor animals are pigs, this complicates the issue for Muslim ethicists [18–20].

In recent times, Dr. Muhammad Mohiuddin— a Muslim physician— has performed xenotransplantation on patients, with some measure of success. Yet, he has encountered some opposition from more traditionalist Muslim religious scholars, who argue that the pig’s impurity renders such procedures *haram.* Prominent scholar Sheikh Mohammed Tarawneh has explained that “if the animal is impure like the pig, the scholars of Islam say it’s haram and it is not permissible to do the transplant if there is an alternative choice” [21]. But he has also added that “if there is no way except through, for example, the transplant from a pig organ… then in that case it takes a ruling of the necessity”, and in that instance, the procedure would be acceptable.

These views seem to be shared by many Muslim religious scholars. For example, scholars Hudzaifah Achmad Qotadah and Maisyatusy Syarifah posit that “the transplantation of a pig’s kidney into a human body is part of the hifdz al nafs effort to implement maqasid al dharuriyyat for the patient’s survival”, and as per Islamic jurisprudence, this is allowed [22]. It must still be noted that a minority of scholars disagree with this assessment [23]. Yet, journalists and sociologists in the Muslim world predict that most people in that region will remain hesitant to the prospect of xenotransplantation. For example, Saad Hasan observes: “It’s highly unlikely that the wider Muslim community will eagerly accept the use of pig organs for human transplant, even if someone’s life is at stake” [24].

Yet, this hypothesis needs to be empirically tested in specific Muslim countries. Some qualitative studies have assessed this issue. For example, one study in Turkey found that “social and religious values, positive, negative and future thoughts” were a recurrent theme in participants’ views on xenotransplantation. However, more thorough, survey-based quantitative studies in Muslim countries are needed to assess people’s attitudes towards xenotransplantation [25]. That is the main objective of this study. We shall therefore attempt to answer the following research questions: To what extent are people in Egypt willing to accept the prospect of xenotransplantation? Would their level of acceptance vary if, instead of a pig, a sheep were used? Egypt has long been the cultural and religious center in the Muslim world, but it has a sizeable Christian population of about 10% [26]. Consequently, it is important to establish if, in that nation, attitudes towards xenotransplantation vary across religious affiliation. The present study establishes that comparison.

Furthermore, religion in Egypt largely serves as a marker of identity, but that is not necessarily the same as level of religiosity itself. It is therefore important to establish if attitudes towards xenotransplantation are also associated with religiosity levels. The present study also tests that hypothesis.

As discussed above, apart from religious issues, the prospect of xenotransplantation elicits moral concerns, to the extent that pigs are harvested for such purposes, and ultimately, they must be killed in the procedures. Animal rights movements have gained influence in recent years [27], so it is also important to establish if attitudes towards animals are associated with approval of xenotransplantation [28]. Pet ownership may also be a relevant factor (to the extent that it may be associated with attitudes towards animals) [29, 30], so the associations amongst these variables are tested in the present study.

Finally, the present study also assesses any association of attitudes of xenotransplantation with conventional demographic variables, such as age, financial status, gender, parenthood and residential zone.

## Methods

### Sampling and data collection

Research protocols were approved by an Institutional Review Board (IRB). Participants were informed about the purpose of research, were assured their answers would remain anonymous, and were informed they could refuse to answer at any time. Participants expressed their informed consent before proceeding to answer the survey.

Power analysis was done to calculate minimum sample size. For a linear regression model with 10 predictors, effect size f^2^ = 0.15, α = 0.05 and power = 0.8, minimum sample size comes out as 118. Sampling was non-probabilistic (on the basis of availability and willingness to respond a survey); inclusion criteria was being a resident of Egypt and fluent in either Arabic or English. Some level of stratification was used in order to assure representativeness on the basis of demographic factors such as age, gender, socio-economic status and educational level; the stratification was done by targeting potential participants with particular demographic characteristics that were anticipated to be minoritarian in the sample (females, non-Muslims, people from rural settings, low socio-economic status). One thousand one hundred eighty-four surveys were sent. Surveys were designed to require complete answers to every question before submission, so no incomplete surveys were returned. Participants scanned a QR code and filled in their responses via an online questionnaire administered through Microsoft Forms. Responses were obtained in November 2023. However, 289 participants were discarded because of wrong answers in attention questions (i.e., questions included to make sure participants are truly paying attention to what is being asked). Consequently, the total sample size was 895.

### Measures and statistical analysis

The survey was composed of 4 parts. The whole questionnaire can be consulted in the supplementary file. First, demographic information was collected along eight variables: 1) Age, 2) gender (Male, Female), 3) Place of residence (Rural, Urban) 4) religion (Islam, Christianity), 5) Pet ownership (Yes, No), 6) Parenthood (Yes, No), 7) completed study level (None, Primary, Secondary, University-Undergraduate, University-Postgraduate; ranked in the variable from 1 to 5), 8) financial status (Poor, Struggling, Stable, Well-secured, Rich, ranked in the variable from 1 to 5).

Second, two questions were asked. The first question was as follows: “In 2021, Dr. Muhammad Mohiuddin successfully implanted a genetically modified pig’s heart into a human cancer patient. The pig died, and the human patient’s life was saved. Do you agree that this was the right thing to do?” This variable was termed “Moral approval of pigs’ organs for xenotransplantation.” The second question was as follows: “Suppose that the same transplant could be done, but instead of using a pig’s organ, a sheep’s organ would be used to save a human patient. The animal would die and the human patient would be saved. Do you agree that this would be the right thing to do?” This variable was termed “Moral approval of sheep’s organs for xenotransplantation.”

Responses to these questions were set on a Likert scale, from 1 (Strongly disagree) to 5 (strongly agree).

Third, the Duke University Religion Index (DUREL) questionnaire was included [31]. This is a 5-item questionnaire that assesses three dimensions of religiosity: organizational religious activity (ORA) (e.g., “How often do you attend mosque/church or other religious meetings?”, non-organizational religious activity (NORA) (e.g., “How often do you spend time in private religious activities, such as prayer, or Scripture study?), and intrinsic religiosity (IR) (e.g., “My religious beliefs are what really lie behind my whole approach to life”). Answers were arranged on Likert scales (1 to 6 and 1 to 5, depending on the item); maximum possible score is 22, minimum possible score is 5. It has been validated across various cultures [32, 33], it has demonstrated strong positive correlations with other measures of religiosity [34], and it is considered very reliable [35]. Higher scores indicated higher levels of religiosity.

Fourth, the Brief Measures of the Animal Attitude Scale (BMAAS) was included. This is a questionnaire that assesses participants’ attitudes towards animals, and it has been previously validated [36]; in previous applications, it has also had good reliability. The questionnaire consists of 10 items (e.g., “It is morally wrong to hunt wild animals for sport”), arranged on Lickert scales, from 1 = strongly disagree to 5 = strongly agree. Maximum possible score is 50, minimum possible score is 1. Some items are reversed scored (e.g., “I do not think that there is anything wrong with using animals in medical research”), and lower scores indicate more demeaning attitudes towards animals. Given that this survey included reversed items, two attention questions were included (“Please select ‘Strongly disagree’ here”, “Please select ‘Strongly agree’ here”), and those participants who did not provide the answers as instructed, were discarded. As mentioned above, 289 participants were discarded on this basis.

Questions were presented simultaneously in English and Arabic; Arabic text was translated back to English in order to screen for errors, no corrections were necessary.

For descriptive statistics, nominal variables were assessed with counts and percentages, and continuous variables were analyzed with mean (*M*) and standard deviation (*S.D.*)*.*

Two main multiple linear regression models were elaborated for inferential statistical analysis. Both regression models include all selected predictors and were presented simultaneously in order to rule our confounding factors, and the tables present the estimates for each predictor, as well as the standardized estimates (i.e., adjusted in order to determine the most significant predictor). Consequently, any associations that turned out to be statistically significant were obtained while controlling for the effect of other predictors.

First, “Moral approval of pigs’ organs for xenotransplantation” was used as the dependent variable, and the following variables were used as predictors: Age, gender, residence, financial status, study level, pet ownership, parenthood, religion, DUREL, BMAAS.

On the basis of statistically significant predictors, a post-hoc regression model was built solely with the significant variables obtained in the prior model.

Second, “Moral approval of sheep’s organs for xenotransplantation” was used as the dependent variable, and the following variables were used as predictors: Age, gender, residence, financial status, study level, pet ownership, parenthood, religion, DUREL, BMAAS.

On the basis of statistically significant predictors, a post-hoc regression model was built solely with the significant variables obtained in the prior model.

The purpose of the post-hoc regression models was to use exclusively the predictors that turned out to be significant in the first place, and therefore make the estimates more precise in the absence of non-significant predictors. This is a procedure that is recommended by some statisticians [37]. For the presentation of results in the post-hoc regression models, any associations that turned out to be statistically significant were obtained while controlling for the effect of other predictors.

In the presentation of the linear regression analyses, results were displayed with the estimates, *p*-values and standardized estimates along the columns, and each of the predictors and the intercept along the rows.

Alpha level was originally placed at *p* ≤ 0.05. However, in order to avoid the multiple comparisons problem, a Bonferroni correction was applied in the original regression models. Therefore, the alpha level was placed at *p* ≤ 0.005 (on the basis of the 10 predictors in the original regression models).

Paired two-tailed t-tests were done comparing responses to “Moral approval of pigs’ organs for xenotransplantation” and “Moral approval of sheep’s organs for xenotransplantation”, for both Muslims and Christians.

Two mediation analyses were done: one for gender as predictor, BMAAS as mediator, and “Moral approval of pigs’ organs for xenotransplantation” as dependent variable; another for gender as predictor, BMAAS as mediator, and “Moral approval of sheep’s organs for xenotransplantation” as dependent variable. Mediation analyses were done following Baron & Kenny’s method [38]. This method goes through three phases: 1) an analysis is done to test if the predictor influences the dependent variable; 2) an analysis is done to test if the predictor influences the mediator; 3) An analysis is done to test if the mediator influences the dependent variable, but also considering the predictor in the regression. Furthermore, a Sobel’s test is run to determine the indirect effect, and if the result turns out statistically significant, we may conclude there is a mediation relationship. Results are presented with β values and *p*-values.

An independent samples two-tailed t-test matrix was built, comparing Christians and Muslims’ responses to each of the statements of the DUREL and BMAAS. In order to avoid the multiple comparisons problem, a Bonferroni correction was applied, and consequently, statistical significance was placed at *p* < 0.003.

Jamovi software was used to calculate descriptive results as well as inferential results. GPOWER was used to calculate the a-priori power analysis for minimum sample size.

## Results

### Descriptive findings

The overall sample size was 895. The sample as a whole was relatively young (*M*_*age*_ = 23.4, *S.D.*_*age*_ *=* 8.57, *range* = 17–66). 93% of respondents were female, 7% were males. 43% of respondents lived in urban settings, 57% lived in rural settings. 88% of respondents owned pets, 12% did not. 91% of respondents did not have children, 9% did. 96% of respondents were Muslims, 4% were Christian. In terms of financial status, 1% described themselves as “poor”, 22% as “struggling”, 59% as “stable”, 17% as well-secured, and 1% as “rich”. In terms of educational level, 0.2% reported no degree completion, 0.1% reported primary level, 41.1% reported secondary level, 51.8% reported university undergraduate level, and 6.7% reported university postgraduate level. Findings for BMAAS were *M*_*BMAAS*_ = 30.7, *S.D.*_*BMAAS*_ = 5.86. Findings for DUREL were *M*_*DUREL*_ = 22.8, *S.D.*_*DUREL*_ = 2.73.

**Fig. 1:**
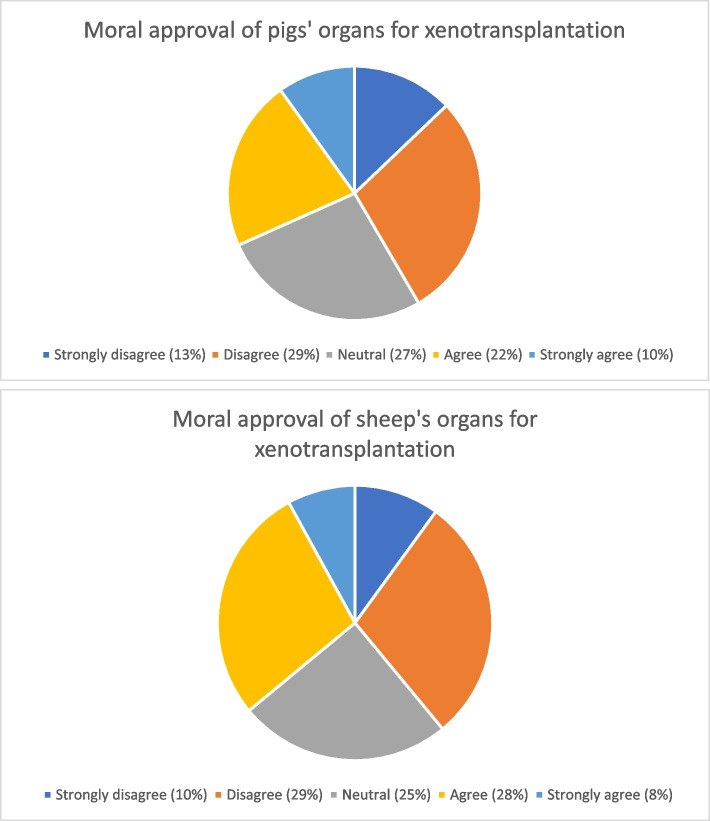
Descriptive plots of moral approval of pigs and sheep as animals for xenotransplantation

In terms of approval of pigs’ organs for xenotransplantation, results were as follows: Strongly disagree = 13%; Disagree = 29%; Unsure = 27%; Agree = 22%; Strongly agree = 10%. When presented as a rank variable, results are *M* = 2.87, *S.D.* = 1.18. In terms of approval of sheep’s organs for xenotransplantation, results were as follows: Strongly disagree = 10%; Disagree = 29%; Unsure = 25%; Agree = 28%; Strongly agree = 8.%. When presented as a rank variable, results were *M* = 2.96, *S.D.* = 1.15. Descriptive plots of these findings are presented in Fig. 1.

When split along the lines of religious identity, the approval of pigs’ organs for xenotransplantation, results were as follows.

For Muslims, in terms of approval of pigs’ organs for xenotransplantation, these were the findings: Strongly disagree = 13%; Disagree = 30%; Unsure = 29%; Agree = 21%; Strongly agree = 9%. When presented as a rank variable, results were *M* = 2.83, *S.D.* = 1.16. In terms of approval of sheep’s organs for xenotransplantation, results were as follows: Strongly disagree = 10%; Disagree = 29%; Unsure = 25%; Agree = 28%; Strongly agree = 8.%. When presented as a rank variable, results were *M* = 2.93, *S.D.* = 1.13.

For Christians, in terms of approval of pigs’ organs for xenotransplantation, these were the findings: Strongly disagree = 9%; Disagree = 9%; Unsure = 9%; Agree = 46%; Strongly agree = 29%. When presented as a rank variable, results were *M* = 3.77, *S.D.* = 1.21. In terms of approval of sheep’s organs for xenotransplantation, results were as follows: Strongly disagree = 9%; Disagree = 9%; Unsure = 11%; Agree = 46%; Strongly agree = 26%. When presented as a rank variable, results were *M* = 3.71, *S.D.* = 1.20.

### Inferential findings

The results of multiple linear regression model with “Moral approval of pigs’ organs for xenotransplantation” as dependent variable is presented in Table 1. The collinearity assumption for this model was met (VIF range = 1.04–1.66; Tolerance range = 0.60–0.99). The model reached a significant level with value of *F*(10,884) = 7.09, *p* < 0.001, and R^2^ value of 0.07. Of the predictors, three showed statistically significant association with “Moral approval of pigs’ organs for xenotransplantation,” including gender, religion and BMAAS. Female gender, Islamic religious identity and higher BMAAS score decreased the likelihood of morally approving the use of pigs for xenotransplantation.

**Table 1:** Multiple linear regression for variable “Moral acceptance of pigs as animals for xenotransplantation”

Dependent variable: Moral acceptance of pigs as animals for xenotransplantation				
Predictor	Estimate	t	*P*	Stand. Estimate
Intercept	3.748	7.691	< 0.001	
Age	0.009	1.510	0.131	0.063
Gender: male (Reference level: female).	0.690	4.098	< 0.001*	0.586
Financial status	0.001	0.030	0.976	9.89E-04
Study level	0.081	1.215	0.225	0.043
Religion: Christianity (Reference level: Islam)	0.589	2.793	0.005*	0.500
Residence: Urban (Reference level: Rural)	−0.140	−1.741	0.082	−0.120
Parenthood: Yes (Reference level: No)	−0.31989	−1.9546	0.051	−0.272
Pet ownership: Yes (Reference level: No)	−0.01357	−0.1132	0.91	−0.011
DUREL	−0.02722	−1.9191	0.055	−0.063
BMAAS	−0.02365	−3.556	< 0.001*	−0.118

*BMAAS* Brief Measures of the Animal Attitude Scale

*DUREL* Duke Religiosity Index

*Statistically significant at *p*-value < 0.05

Based on these three significant predictors, a post-hoc regression model was built. Gender significantly predicted moral approval of pigs’ organs for xenotransplantation, β = 0.59, t(891) = 4.51, *p* < 0.001; religion significantly predicted moral approval of pigs’ organs for xenotransplantation, β = 0.58, t(891) = 3.33, *p* < 0.001; BMAAS significantly predicted moral approval of pigs’ organs for xenotransplantation, β = − 0.11, *t*(891) = − 3.38, *p* < 0.001. Consequently, the results suggest the strongest predictor was gender, followed by religion, followed by BMAAS. The post-hoc regression analysis yielded similar results as the linear regression analysis and therefore, this suggests a validation of the overall analysis.

The results of multiple linear regression model with “Moral approval of sheep’s organs for xenotransplantation” as dependent variable is presented in Table 2. Collinearity assumption for this model was met (VIF range = 1.04–1.66; Tolerance range = 0.60–0.99). The model reached a significant level with value of *F*(10,884) = 9.99, *p* < 0.001, and R^2^ value of 0.1. Of the predictors, two showed statistically significant association with “Moral approval of sheep’s organs for xenotransplantation,” including gender and BMAAS. Female gender, Islamic religious identity and higher BMAAS score decreased the likelihood of morally approving the use of pigs for xenotransplantation. Female gender and higher BMAAS score decreased the likelihood of morally approving the use of sheep for xenotransplantation.

**Table 2:** Multiple linear regression for variable “Moral acceptance of sheep as animals for xenotransplantation”

Dependent variable: Moral acceptance of sheep as animals for xenotransplantation				
Predictor	Estimate	T	*P*	Stand. Estimate
Intercept	3.82	8.18	< 0.001	
Age	0.01	1.56	0.12	0.06
Gender: male (Reference level: female).	0.79	4.89	< 0.001*	0.69
Financial status	0.03	0.62	0.53	0.02
Study level	0.07	1.13	0.26	0.04
Religion: Christianity (Reference level: Islam)	0.33	1.62	0.11	0.29
Residence: Urban (Reference level: Rural)	−0.14	−1.86	0.06	−0.13
Parenthood: Yes (Reference level: No)	−0.10	−0.63	0.53	−0.09
Pet ownership: Yes (Reference level: No)	0.15	1.32	0.19	0.13
DUREL	−0.02	−1.35	0.18	−0.04
BMAAS	−0.03	−5.11	< 0.001*	−0.17

*BMAAS* Brief Measures of the Animal Attitude Scale

*DUREL* Duke Religiosity Index

*Statistically significant at *p*-value < 0.05

On the basis of these two significant predictors, a post-hoc regression model was built. Gender significantly predicted moral approval of sheep’s organs for xenotransplantation, β = 0.88, t(892) = 7.09, *p* < 0.001; BMAAS significantly predicted moral approval of pigs’ organs for xenotransplantation, β = − 0.15, t(892) = − 4.74, *p* < 0.001. Consequently, the results suggest the strongest predictor was gender, followed by BMAAS. The post-hoc regression analysis yielded similar results as the linear regression analysis and therefore, this suggests a validation of the overall analysis.

Amongst Christians, there was no significant difference between “Moral approval of pigs’ organs for xenotransplantation” and “Moral approval of sheep’s organs for xenotransplantation”, *t*(34) = 1, *p* = 0.32. Amongst Muslims, there was a significant difference between “Moral approval of pigs’ organs for xenotransplantation” and “Moral approval of sheep’s organs for xenotransplantation”, *t*(859) = − 2.97, *p* = 0.003, with a small effect size, *d =* − 0.1.

BMAAS did not mediate the relationship between gender and “Moral approval of pigs’ organs for xenotransplantation”, as the indirect effect was not significant as per Sobel’s test (z = 1.91, *p* = 0.06). Results of this moderation analysis are presented in Table 3.

**Table 3:** Mediation analysis. Dependent variable: “Moral acceptance of pigs as animals for xenotransplantation.” Mediator: BMAAS. Predictor: gender

Effect	Estimate	Z	*P*
Indirect	0.04	1.91	0.05
Direct	0.85	5.66	< 0.001*
Total	0.88	5.90	< 0.001*

*Statistically significant at *p*-value < 0.05

BMAAS partially mediated the relationship between gender and “Moral approval of sheep’s organs for xenotransplantation.” The rationale for this finding met the three criteria stipulated by Baron and Kenny’s method of three regressions. First, gender predicted “Moral approval of sheep’s organs for xenotransplantation” (β = 0.24, *p* < 0.001); second, gender predicted BMAAS (β = − 0.08, *p* = 0.02); third, when adding BMAAS gender still predicted “Moral approval of sheep’s organs for xenotransplantation”, but at a lower strength (β = 0.207, *p* < 0.001), and BMAAS predicted “Moral approval of sheep’s organs for xenotransplantation” (β = − 0.15, *p* < 0.001). Furthermore, the indirect effect as per Sobel’s test was significant (z = 2.11, *p* = 0.04). The results of this mediation are presented in Table 4.

**Table 4:** Mediation analysis. Dependent variable: “Moral acceptance of sheep as animals for xenotransplantation.” Mediator: BMAAS. Predictor: gender

Effect	Estimate	Z	*P*
Indirect	0.05	2.11	0.04*
Direct	1.01	7.10	< 0.001*
Total	1.07	7.41	< 0.001*

*Statistically significant at *p*-value < 0.05

Matrix of t-tests comparing Muslims and Christians’ responses to each statement of DUREL and BMAAS are presented in Table 5. Muslims have significant higher scores in the following items: “How often do you spend time in private religious activities, such as prayer, or Scripture study?” (DUREL); “In my life, I experience the presence of the Divine (i.e., God)” (DUREL); “I try hard to carry my religion over into all other dealings in life” (DUREL); Christians have significant higher score in this item: “How often do you attend mosque/church or other religious meetings? (DUREL)”.

**Table 5:** T-test matrix for each item of the DUREL and BMAAS. Dependent variable: “Religion”

Item	Muslims mean score	Christians mean score	T-statistic	*P*-value	Effect size (Cohen’s d)
How often do you attend mosque/church or other religious meetings? (DUREL)	3.38	4.46	−3.37	< 0.001*	−0.58
How often do you spend time in private religious activities, such as prayer, or Scripture study? (DUREL)	4.83	3.4	6.71	< 0.001*	1.16
In my life, I experience the presence of the Divine (i.e., God) (DUREL)	4.97	4.46	9.17	< 0.001*	1.58
My religious beliefs are what really lie behind my whole approach to life (DUREL)	4.57	4.26	1.93	0.05	0.33
I try hard to carry my religion over into all other dealings in life (DUREL)	4.87	4.31	7.01	< 0.001*	1.21
It is morally wrong to hunt wild animals for sport (BMAAS)	3.14	3.20	−0.21	0.83	−0.04
I do not think that there is anything wrong with using animals in medical research (BMAAS)	2.50	2.26	1.03	0.30	0.18
I think it is perfectly acceptable for cattle and sheep to be raised for human consumption (BMAAS)	1.48	1.80	−1.99	0.05	−0.34
Basically, humans have the right to use animals as we see fit (BMAAS)	3.87	3.49	1.65	0.10	0.29
The slaughter of whales and dolphins should be immediately stopped even if it means some people will be put out of work (BMAAS)	3.52	3.83	−1.27	0.20	−0.22
I sometimes get upset when I see wild animals in cages at zoos (BMAAS)	3.56	3.71	−0.67	0.51	−0.12
Breeding animals for their skins is a legitimate use of animals (BMAAS)	2.86	2.54	1.26	0.21	0.22
Some aspects of biology can only be learned through dissecting preserved animals such as cats (BMAAS)	2.94	2.40	2.19	0.03	0.38
It is unethical to breed purebred cats when millions of cats are killed in animal shelters each year (BMAAS)	3.25	3.69	−1.57	0.12	−0.27
The use of animals such as rabbits for testing the safety of cosmetics and household products is unnecessary and should be stopped (BMAAS)	3.60	3.97	−1.46	0.15	−0.25

* Statistically significant value at *p* < 0.003

## Discussion

This study indicates that people’s views on xenotransplantation in Egypt are not dramatically different from those held in other countries [39–42]. While the benefits of xenotransplantation are appreciated, there may still be some ethical concerns, and this is reflected in the overall moral approval rate. The results of the present study suggest that moral opinions about this issue are roughly evenly distributed, suggesting that it is a complicated moral matter.

The prospect of xenotransplantation makes some people uneasy, given that it raises ethical questions about the boundaries between species [43–47]. This concern typically has various dimensions. First, there is the issue of human exceptionalism. Many philosophical and religious traditions emphasize the unique status of humans. The idea of human exceptionalism asserts that humans possess a distinct moral, intellectual, or spiritual essence that sets them apart from other species. The crossing of species boundaries, such as through genetic engineering or xenotransplantation, challenges this perception and raises ethical questions about the manipulation of fundamental aspects of life. Ethical frameworks often draw on distinctions between species to guide moral decision-making. Some individuals believe that certain rights and responsibilities are exclusive to humans, based on perceived moral or cognitive differences. The blurring of species boundaries, as in the case of xenotransplantation, can provoke ethical dilemmas for those who hold such beliefs.

Some authors call this the “xenotransplantation paradox.” Gill Haddow defines it as the tension in thinking that “although non-human animals and humans are thought to be biologically compatible or similar, many assume and emphasise just how different we are from non-human animals…The paradox of xenotransplantation is one that simultaneously highlights how deep the need is for a natural boundary to exist between what is human and what is a non-human animal and yet how shallow the socially constructed division between the species is” [48]. From a moral psychology perspective, xenotransplantation elicits cognitive discomfort in many people, to the extent that animals used as donors must be sufficiently close to humans (for purposes of anatomical and physiological compatibility, etc.), yet sufficiently distant to not be disturbed by their exploitative use.

Likewise, there is a fear among some individuals that manipulating species boundaries reflects human hubris [49–52], an excessive pride or arrogance that could lead to unintended consequences. The idea that humans should respect the natural order and not overstep their role in the ecosystem is rooted in environmental ethics and concerns about ecological balance. Furthermore, some individuals and groups advocate for environmental stewardship, arguing that respecting species boundaries is essential for maintaining biodiversity and ecological balance. The fear is that interventions that blur these boundaries might disrupt ecosystems and have unforeseen consequences on a broader scale. There is also a natural human tendency to be cautious and even fearful of the unknown. Manipulating species boundaries often involves venturing into uncharted territories in terms of both science and ethics. This fear of the unknown can lead to skepticism or outright opposition to activities that challenge established boundaries [53, 54].

These ethical concerns are of a secular nature, but it is important to determine if religious objections play any role in the ethical approach to xenotransplantation in Egypt. The results of the present study suggest that religiosity itself plays no role, but religious identity does. It is important to distinguish both concepts, while admitting that there is some fluidity and overlap between them [55–57]. Religiosity primarily focuses on an individual’s personal beliefs, practices, and spiritual experiences; in contrast, religious identity focuses on the social and cultural aspects of affiliation with a religious group and the outward expression of that affiliation. Religiosity can vary in intensity, with individuals exhibiting different levels of commitment to religious beliefs and practices; in contrast, religious identity involves a stable sense of belonging to a particular religious group, even if the intensity of religious commitment may vary.

As per the regression models of this study, the level of religiosity has no bearing on participants’ moral approval of xenotransplantation with either pigs or sheep. But based on this study (admittedly with a small Christian proportion not entirely reflective of the Egyptian distribution of religious identity) it seems Christian identity is associated with a greater level of acceptance of xenotransplantation in comparison to Muslim identity. However, this tentative difference in moral approval of xenotransplantation is only present when the animal for xenotransplantation is the pig. This finding is expected, to the extent that Islam stipulates religious prohibitions against the consumption of pork. Therefore, Muslims and Christians in this study seem to hold different views about the ethical value of xenotransplantation only when it comes to pigs.

Interestingly, amongst the Christians of this study (again, it is important to bear in mind that the number of Christians in the study was small as compared to the proportion in the Egyptian population at large), the choice of animals makes no difference in moral approval of xenotransplantation. But, as per the paired t-test, amongst Muslims there is a significant difference between approval of xenotransplantation when the animal is a pig, and approval of xenotransplantation when the animal is a sheep. There is greater approval of sheep as donors, although admittedly, the effect size is small. Ultimately, then, the Islamic religious prohibition on pigs does have an effect on Egyptians’ moral views about xenotransplantation. But interesting, this religious aspect applies not properly to the prospect of xenotransplantation itself, but rather, to the use of pigs as donors.

While such prohibitions are explicitly stated in the Qur’an (as mentioned above), amongst Muslims there may be various religious rationales [58, 59]. Pigs are known to carry various diseases and parasites, some of which can be transmitted to humans. In the historical context of seventh-century Arabia when Islam emerged, there was limited knowledge about proper sanitation and the transmission of diseases. The prohibition on pork is seen, in part, as a measure to protect the health of the community. The prohibition of pork may not only be about physical health but also carries symbolic and spiritual significance. Following dietary restrictions, including abstaining from pork, may be considered a test of obedience and submission to God’s commandments. Many Muslims believe that adhering to these prohibitions is a way of demonstrating faith and commitment to the teachings of Islam [60, 61].

In this context, it is important to consider how religious identity and secularization have interacted in Egypt in recent history [62–64]. Egypt experienced British and French colonial rule in the 19th and early 20th centuries. This period brought with it the introduction of Western ideas, institutions, and educational systems that were often more secular in nature [65, 66]. The colonial experience contributed to the erosion of traditional religious authority and the rise of more secular institutions. The promotion of education, particularly in the modern sciences and technologies, has played a role in secularization. As educational systems have expanded, there has been an increased emphasis on rational and empirical approaches, often sidelining religious doctrines in certain spheres. Legal reforms have been implemented to modernize and secularize legal systems in Egypt. Family laws, for example, have undergone changes to be more in line with modern legal principles, sometimes diverging from traditional Islamic legal interpretations. Furthermore, social and economic changes, including the rise of the middle class and changes in work patterns, can influence the way people prioritize their lives. As societies become more economically developed, there is often a decrease in the reliance on traditional religious structures for social and economic support [67–69].

Yet, this secularization process has also been balanced with adherence to a Muslim identity. Religion continues to play a prominent role in Egyptian public life (in the realms of public prayer, family values, religious instructions, cultural heritage, and the prominence of Islamic media, amongst others) [70–73]. Furthermore, Islamic organizations and charities play a role in community development, social services, and humanitarian work [74–77].

It is interesting to note that the results of the present study suggest that secularization has had different effects when comparing Christians and Muslims in Egypt. For example, Christians are more likely to visit temples than Muslims. One possible explanation is that, given their minority status in Egypt, Christians feel the need to find a meeting place with coreligionists on a regular basis, whereas Muslims do not, since Egyptian society at large is dominated by Muslim life. On the other hand, Muslims spend more time in prayer and other religious activities, Muslims are more likely to have more religious experiences in daily life and are more likely to carry religion into every aspect of their lives.

Ultimately, this tension between religious identity and secularization may have an effect on Egyptians’ approach to xenotransplantation. The results of the present study suggest that Muslim Egyptians may be more open to morally approve of xenotransplantation if an animal not considered impure would serve as donor. While the use of animals considered pure for consumption in Islam (e.g., sheep, goats, cows) are more difficult to work with in the prospect of xenotransplantation, it is important for researchers to understand that given the demographic weight of Muslims around the world, there may be some need to develop research in xenotransplantation with such animals, to increase acceptance of xenotransplantation in Muslim countries such as Egypt.

As in many other countries, secularization in Egypt has been more intense in urban environments [78, 79]. Yet, the results of the present study indicate that the place of residence in Egypt (countryside vs. cities) is not associated with moral approval of xenotransplantation. Likewise, it may be presumed that younger people are more open to technological innovations and are less sensitive to the strictness of religious identity and religious prohibitions [80–82]. Yet, the results of the present study also indicate that age is not a predictive factor of moral approval of xenotransplantation, regardless of the animal.

Likewise, secularization in most contexts (including Egypt) has been associated with higher educational [83–85] and socio-economic levels [86, 87]. However, in this study, neither of those variables predict moral approval of xenotransplantation.

It is important to consider that, while religious identity is a factor in predicting the moral approval of xenotransplantation, attitudes towards animals (as measured by the BMAAS) are also a factor, albeit in a lower capacity. Indeed, as per the regression model of this study, greater attitudes of respect towards animals predict lower moral approval of xenotransplantation.

This is expected, as the prospect of xenotransplantation raises concern about the welfare of animals. The genetic modification of animals for xenotransplantation purposes can involve altering their physiology to reduce the risk of organ rejection in humans; this may have a potential impact on the health and natural behaviour of the animals. Since xenotransplantation involves the sacrifice of animals for the benefit of humans, people who feel more empathy towards animals would likely oppose xenotransplantation. Subjects with a more elevated sense of the need for animal welfare would contend that animals should not be treated as mere resources for human use and that their interests, including their right to live free from unnecessary harm, should be respected.

While Egypt has not been traditionally at the forefront of animal rights activism, movements have been on the rise in recent years [88], and there have been some governmental initiatives in this regard [89]. Consequently, although not to the same level as religious identity, animal welfare concerns are a predictive factor of moral approval of xenotransplantation, and policymakers and researchers seeking to establish a firmer hold of xenotransplantation in this nation, need to seriously consider ways of ensuring greater measures of animal welfare in xenotransplantation procedures, to persuade people to morally approve of this technology.

However, it is important not to overestimate the role of religious identity or attitudes towards animals (as measured by the BMAAS) in the moral approval of xenotransplantation. For, as per the results of the regression model in the present study, the strongest predictor of moral approval for xenotransplantation (regardless of the animal) was gender, with males having a greater rate of approval.

Previous research has established that there are some gender differences when it comes to moral reasoning. Following Kohlberg’s moral development stages, some research suggests that women tend to reach higher stages of moral reasoning earlier than men [90, 91]. Likewise, some research suggests that women often approach moral dilemmas with an “ethic of care,” emphasizing relationships, empathy, and concern for others, whereas men may lean more toward an “ethic of justice,” focusing on principles, rights, and fairness [92].

Studies also suggest that women, on average, may demonstrate higher levels of empathy and compassion compared to men [93–95]. This emotional responsiveness can influence moral reasoning, leading to considerations of the impact of actions on others and a tendency toward caring orientations. Likewise, some research indicates that women may be more sensitive to issues related to harm and care, showing a greater concern for avoiding harm to others [96, 97]. This sensitivity may influence moral decision-making, particularly in situations where potential harm is a central ethical consideration.

In the present study, women were less likely to morally approve of xenotransplantation. One possible mediating factor may be attitudes towards animals (as measured by the BMAAS). As indicated above, in this study greater concern for animal welfare predicts reduced moral approval of xenotransplantation. As per the results of the regression model (as part of the mediation analysis), gender also predicts higher concern for animal welfare, with women expressing more caring attitudes towards animals (as measured by the BMAAS).

Indeed, this reproduces some findings of other studies [98–100]. Previous research has established that women, on average, may exhibit higher levels of empathy and compassion toward animals compared to men. Likewise, women are often prominent in animal advocacy and activism; they may be more likely to engage in activities such as volunteering at animal shelters, supporting animal rights organizations, and advocating for humane treatment. Women, on average, may be more likely to own pets and to express strong emotional bonds with their animals; the companionship and caregiving aspects of pet ownership can contribute to a heightened awareness of animal welfare issues [101]. Women are often overrepresented in occupations related to animal care, such as veterinary medicine, animal welfare organizations, and pet grooming [102]; this professional involvement may contribute to a heightened awareness of and concern for animal welfare.

As per the mediation analysis of this study, attitudes towards animals (as measured by the BMAAS) serve as partial mediator of women’s greater moral disapproval of xenotransplantation. Therefore, once again, assuming that xenotransplantation is a worthy moral endeavor (to the extent that, as mentioned in the Introduction, xenotransplantation is a viable partial solution to the problem of shortage of organs in donation), and assuming that it is desirable to increase the moral acceptance of xenotransplantation amongst Egyptians, policymakers and researchers ought to focus on women. But in so doing, it must be understood that an important way of increasing women’s approval of xenotransplantation comes through some assurance that animals used in xenotransplantation would be offered some acceptable measure of welfare.

Interestingly, in this study, pet owners do show attitudes of greater care towards animals, as measured by BMAAS (*t*(893) = − 3.59, *p* < 0.01, *d* = − 3.67). This reproduces some results in previous research [103, 104]. But in this study, owning a pet is not, by and of itself, a relevant factor in predicting the moral approval of xenotransplantation. The relevant aspect is attitudes towards animals (as measured by the BMAAS) on a more abstract level.

Finally, it is important to consider whether having a child may predict approval of xenotransplantation. Previous research has shown that being a parent may be associated with greater sensitivity towards human suffering [105–107]. Consequently, it is plausible to argue that, to the extent that xenotransplantation saves human lives, parents are more likely to approve of it. However, no such results have been obtained in the present study, and further research in other cultural contexts is needed in order to determine if there is any association between parenthood and moral approval of xenotransplantation.

To sum up, the original research questions of this article can be answered as follows. First, to what extent are people in Egypt willing to accept the prospect of xenotransplantation? Answer: results in Egypt are fairly similar to other countries, especially in the MENA region. Second: do attitudes towards xenotransplantation vary across religious affiliation? Answer: yes, in light of the results, they do, Christians are more likely to have greater moral approval of xenotransplantation. Third: are attitudes towards xenotransplantation also associated with religiosity levels? Answer: no, they are not, considering the results of the present study. Fourth: Are attitudes towards animals associated with moral approval of xenotransplantation? Answer: results suggest that, yes, they are, the higher the level of positive attitudes towards animals (as measured by the BMAAS), the lower the moral approval of xenotransplantation. Fifth: are demographic variables, such as age, financial status, gender, parenthood, pet ownership and residential zone, associated with moral approval of xenotransplantation? Answer: results suggest that only gender is associated with moral approval of xenotransplantation, and women express lower levels of approval.

### Limitations

This study’s sample was sufficiently large to achieve an acceptable level of statistical power. However, the sampling was non-probabilistic (on account of logistics to recruit participants) and did not entirely reflect the gender and religious identity composition of Egypt; in this study, women and Muslims were overrepresented, and this may have rendered the sampling less representative of the population at large. We acknowledge this disparity as a limitation; however, in the case of gender, previous research has established that as a general trend, women are more receptive to answer surveys [108]. Given that men and Christians were not sufficiently represented (i.e., their proportion in the sample was smaller than their proportion in the Egyptian population at large), the results in comparisons with women and Muslims, respectively, may have been skewed. Further studies that rely on more stratified sampling may be necessary to arrive at more robust conclusions.

## Conclusion

Xenotransplantation is a clinical biotechnology that has great potential to alleviate the global shortage of organs available for transplantation. However, whilst conventional organ donation involves several ethical questions, xenotransplantation raises several additional novel ethical objections.

This may be especially the case in the Muslim world, as the animal most suited for xenotransplantation is the pig, and this animal has traditionally been considered impure in Islam. The results of the present study indicate that, indeed, the fact that xenotransplantation involves the use of pig tissue is an obstacle to moral approval amongst Muslims in Egypt. But importantly, the relevant factor is not the level of religiosity itself, but rather, religious identity. Muslims in Egypt seem to be less likely than Christians to morally approve of xenotransplantation, but only when it involves pigs. Likewise, Muslims in Egypt are more likely to accept xenotransplantation if it involves sheep’s organs. However, it is important to keep in mind that this study is limited to the extent that the proportion of Christian participants was small (lower than the proportion of Christians in Egypt at large), and therefore, no overly robust conclusions can be obtained.

It is important to emphasize that the present study is concerned with moral psychology, and it is not a normative treatise. We have sought to examine what participants think about the ethics of xenotransplantation, but we have not sought to examine whether such stances are morally correct or not. Indeed, the ethics of xenotransplantation may be a nonstarter, to the extent that from the onset, such a project may be morally flawed. Even if a particular animal that is not considered impure in any major religion is chosen (say, the sheep) for xenotransplantation, even if we assume it to be ethically acceptable to blur the boundaries between species by creating human-animal hybrids, and even if animal donors are given good treatment and are painlessly killed to procure their organs, some philosophers may make the case that xenotransplantation is still immoral.

Philosophers have long debated whether it is immoral to eat meat. A growing number of ethicists claim that it is *not* moral to eat meat, to the extent that animals are sentient beings (as Bentham famously asserted, “the question is not, Can they reason?, nor Can they talk? but, Can they suffer?” [109], and furthermore, meat is not a true necessity for human diet. Another line of reasoning (famously advanced by ethicist Tom Regan [110]) posits that even if the animals are killed painlessly (as may be the case in xenotransplantation), it is still immoral to do so, to the extent that animals have rights, and killing them for the purpose of human exploitation violates their right to life.

But xenotransplantation is in one dimension different from eating meat, as this technological prospect may indeed be a necessity for the survival of terminally ill people. In this case, xenotransplantation may be more akin to animal experimentation, to the extent that it is a procedure that entails the killing of animals in order to save human beings. Philosophers also debate the ethics of animal experimentation. Some ethicists (such as Gary Francione [111]) think that animal experiments are never ethically permissible; others (such as Carl Cohen [112]) think we should prioritize human suffering above animal suffering (since animals do not have the same moral status, given their limited capacity for moral duties), and therefore, experiments are permissible; yet others (such as Peter Singer in his more recently revised views) are overall very skeptical of the moral permissibility of animal experimentation, but remain open to the possibility, provided the benefits truly outweigh the harms, considering all sentient beings involved in the procedures [113].

While ethical discussions on animal experimentation have been happening for quite some time, xenotransplantation presents a new challenge. For now, it is difficult to predict what the eventual outcome of this ethical discussion will be. But if ethicists eventually reach a consensus that xenotransplantation is a morally acceptable procedure (admittedly, that is a big “if”) and it is one that is worth pursuing in biotechnological advancement, then the present study has some implications for policy.

On the one hand, for xenotransplantation to be more accepted in Muslim countries, researchers need to start considering alternatives to pigs in procedures and explore the possibility of using permissible animals in Islam (sheep, cows, etc.). Admittedly, this is a very difficult endeavor, as there are many technical problems in using these animals for xenotransplantation.

On the other hand, assuming that xenotransplantation becomes an effective and safe procedure, policymakers need to work with religious authorities to favor an interpretation of Islam in which pigs are not allowed for consumption, but they may be allowed as sources of organ transplantation, especially considering that xenotransplantation may save lives, and Islam has a strong tradition of flexibility if it comes to life-saving procedures.

Furthermore, it is important to highlight that religious identity (not religiosity) is the relevant factor in predicting moral approval of xenotransplantation, and consequently, policymakers ought to work with religious and secular authorities— again, assuming the full viability and success of xenotransplantation— in order to find a way of assuring Muslims (regardless of their level of religiosity) that xenotransplantation from pigs is not a threat to their religious identity.

However, it is more likely that the core of xenotransplantation (and not merely its accessory features) will remain ethically contentious. In that case, it is important for leaders of civil society in Egypt to facilitate discussions in order to help people make informed decisions that align with their moral interests, and on that basis, allow people to come to their own moral conclusions regarding xenotransplantation. If some people come to decide that there is no scenario in which xenotransplantation is morally admissible, then that commitment should be respected. If some other people come to decide that xenotransplantation as a concept may be morally acceptable, then promoters of xenotransplantation must dedicate substantial efforts to meet the ethical guidelines for the proper treatment of animals in xenotransplantation, as this may prove to be an effective strategy in persuading people in Egypt to offer greater moral approval of xenotransplantation. Of course, it is sensible to posit that good animal treatment should be implemented for its own sake, but promoters of xenotransplantation should understand that the quality of animal treatment is fundamental in preserving the base of people who morally approve of xenotransplantation, as bad animal treatment would likely turn away people from approving xenotransplantation procedures, and women are more sensitive on this issue.

### Supplementary Information

**Supplementary material 1.**

## Data Availability

The datasets generated during and/or analyzed during the current study are available from the corresponding author on reasonable request.
