# Supplementary material for: Moral approval of xenotransplantation in Egypt: associations with religion, attitudes towards animals and demographic factors
Source: BMC Med Ethics. 2024 Feb 19;25:19. doi: 10.1186/s12910-024-01013-3 (PMC10877808; doi:10.1186/s12910-024-01013-3)
Supplement: Supplementary file 1 — Supplementary material 1. [file 12910_2024_1013_MOESM1_ESM.docx]

**Moral approval of xenotransplantation in Egypt:**

**associations with religion, attitudes towards animals and demographic factors**

**Gabriel Andrade^1^**

**Eid Abo Hamza^2^**

**Yasmeen Elsantil^3^**

**Alaa Eldin A. Ayoub^4^**

**Dalia Bedewy^5^***

**1. Ajman University, Ajman, United Arab Emirates**

**2. Al Ain University, Al Ain, United Arab Emirates**

**3. Tanta University, Tanta, Egypt**

**4. Arabian Gulf University, Manama Bahrein; Aswan University, Aswan, Egypt**

**5. Ajman University, Ajman, United Arab Emirates; Tanta University, Tanta, Egypt**

**Corresponding author email: d.bedewy@ajman.ac.ae**

**Xenotransplantation Questionnaire**

These responses are anonymous, and you can refuse to answer at any time. Do you agree to proceed?

Yes

What is your age?

What is your gender?

Male

Female

Residence:

City

Rural

How would you describe your financial status:

- Poor
- Struggling
- Stable
- Well- secured
- Rich

Do you own pets (cats, dogs, etc.)?

Yes

No

Do you have children?

Yes

No

What is your religion?

Islam

Christianity

What is your completed study level?

None

Primary

Secondary

University-Undergraduate

University-Postgraduate

In 2021, Dr. Muhammad Mohiuddin successfully implanted a genetically modified pig’s heart into a human cancer patient. The pig died, and the human patient’s life was saved. Do you agree that this was the right thing to do?

A Strongly agree

B Agree

C Unsure

D. Disagree

E. Strongly disagree

Suppose that the same transplant could be done, but instead of using a pig’s organ, a sheep’s organ would be used to save a human patient. The animal would die and the human patient would be saved. Do you agree that this is the right thing to do?

A Strongly agree

B Agree

C Unsure

D. Disagree

E. Strongly disagree

Duke University Religiosity Index:

<https://doi.org/10.3390/rel1010078>

1. How often do you attend mosque/church or other religious meetings?

a – Never

b - Once a year or less

c - A few times a year

d - A few times a month

e - Once a week

f - More than once/week

2. How often do you spend time in private religious activities, such as prayer, or Scripture study?

a - Rarely or never

b - A few times a month

c - Once a week

d - Two or more times/week

e – Daily

f - More than once a day

The following section contains 3 statements about religious belief or experience. Please mark the extent to which each statement is true or not true for you.

3. In my life, I experience the presence of the Divine (i.e., God)

A. Definitely not true

B. Tends not to be true

C. Unsure

D. Tends to be true

E. Definitely true of me

4. My religious beliefs are what really lie behind my whole approach to life

A. Definitely not true

B. Tends not to be true

C. Unsure

D. Tends to be true

E. Definitely true of me

5. I try hard to carry my religion over into all other dealings in life

A. Definitely not true

B. Tends not to be true

C. Unsure

D. Tends to be true

E. Definitely true of me

Brief Measures of the Animal Attitude Scale.

DOI: <https://doi.org/10.2752/089279315X14129350721894>

|  | Strongly agree | Agree | Unsure | Disagree | Strongly disagree |
| --- | --- | --- | --- | --- | --- |
| It is morally wrong to hunt wild animals for sport |  |  |  |  |  |
| I do not think that there is anything wrong with using animals in medical research |  |  |  |  |  |
| I think it is perfectly acceptable for cattle and sheep to be raised for human consumption |  |  |  |  |  |
| Basically, humans have the right to use animals as we see fit |  |  |  |  |  |
| The slaughter of whales and dolphins should be immediately stopped even if it means some people will be put out of work |  |  |  |  |  |
| I sometimes get upset when I see wild animals in cages at zoos |  |  |  |  |  |
| Breeding animals for their skins is a legitimate use of animals |  |  |  |  |  |
| Some aspects of biology can only be learned through dissecting preserved animals such as cats |  |  |  |  |  |
| Please select “Strongly agree” here |  |  |  |  |  |
| It is unethical to breed purebred cats when millions of cats are killed in animal shelters each year |  |  |  |  |  |
| The use of animals such as rabbits for testing the safety of cosmetics and household products is unnecessary and should be stopped |  |  |  |  |  |
| Please select “Strongly disagree” here |  |  |  |  |  |
